# Racial Differences in the Rate of Change in Anterior Lamina Cribrosa Surface Depth in the African Descent and Glaucoma Evaluation Study

**DOI:** 10.1167/iovs.62.4.12

**Published:** 2021-04-12

**Authors:** Christopher A. Girkin, Akram Belghith, Christopher Bowd, Felipe A. Medeiros, Robert N. Weinreb, Jeffrey M. Liebmann, James A. Proudfoot, Linda M. Zangwill, Massimo A. Fazio

**Affiliations:** 1 Department of Ophthalmology and Visual Science, School of Medicine, The University of Alabama at Birmingham, Birmingham, Alabama, United States; 2 Hamilton Glaucoma Center, Shiley Eye Institute, Viterbi Family Department of Ophthalmology, University of California San Diego, La Jolla, California, United States; 3 Duke Eye Center and Department of Ophthalmology, Duke University, Durham, North Carolina, United States; 4 Bernard and Shirlee Brown Glaucoma Research Laboratory, Edward S. Harkness Eye Institute, Columbia University Medical Center, New York, New York, United States; 5 Department of Biomedical Engineering, School of Engineering, The University of Alabama at Birmingham, Birmingham, Alabama, United States

**Keywords:** optic nerve, glaucoma, lamina cribrosa, optical coherence tomography

## Abstract

**Purpose:**

The purpose of this study was to determine if the rate of change in the depth of the surface of the lamina cribrosa due to glaucomatous remodeling differs between glaucoma patients of African descent (AD) and European descent (ED).

**Methods:**

There were 1122 images taken longitudinally over an average of 3 years (range = 0.9–4.1 years) from 122 patients with glaucoma followed in the African Descent and Glaucoma Evaluation Study (ADAGES) and Diagnostic Intervention and Glaucoma Study (DIGS) were automatically segmented to compute anterior lamina cribrosa surface depth (ALCSD). The rate of ALCSD change was compared across racial groups after adjusting for baseline characteristics known to be associated with ALCSD or disease progression (visual field, ALCSD, corneal thickness, optic disk size, and age).

**Results:**

After adjusting for all other covariates, the ED group had significantly greater ALCSD posterior migration (deepening) than the AD group (difference = 2.57 µm/year, *P* = 0.035). There was a wider range of ALCSD change in the ED compared with the AD group, and more individuals had greater magnitude of both deepening and shallowing. No other covariates measured at baseline had independent effects on the longitudinal changes in ALCSD (baseline visual field severity, baseline ALCSD, corneal thickness, Bruch's membrane opening [BMO] area, or age).

**Conclusions:**

Glaucomatous remodeling of the lamina cribrosa differs between AD and ED patients with glaucoma. Unlike the cross-sectional associations seen with aging, in which a deeper ALCSD was seen with age in the ED group, glaucomatous remodeling in this longitudinal study resulted in more posterior migration of ALCSD in ED compared to AD patients.

Spectral-domain optical coherence tomography (SD-OCT) imaging has enabled visualization and structural quantification of the underlying load bearing connective tissues of the optic nerve head (ONH) distinct from the overlying neurovascular tissues they support.^1^ Remodeling of these tissues is a hallmark of glaucomatous optic neuropathy (GON) and is driven by numerous inter-related factors that may modulate ONH mechanical strain, including the tissue's three-dimensional histomorphology, material properties, and perfusion.^2^ A few longitudinal studies in Asian^3^^,^^4^ and European-derived^5^ populations have shown that alterations in the lamina cribrosa in GON are visualized with SD-OCT, including posterior and anterior migration of the lamina cribrosa within the neural canal.^3^^–^^5^

We have previously demonstrated that the mechanical behavior of the healthy ONH in response to IOP changes differs between individuals of African descent (AD) and European descent (ED).^6^ Because mechanical response is a key driver of remodeling of load bearing tissues,^7^^–^^17^ it is likely that variation in mechanical responses may result in differences in remodeling of these tissues due to aging and glaucoma.^18^ Indeed, it appears, using cross-sectional data, that these differences in mechanical behavior may result in the differential changes in anterior lamina cribrosa surface depth (ALCSD) seen with aging across these two racial groups.^19^ If there is a differential response to the remodeling of these load bearing tissues in glaucomatous injury, this may explain, in part, the greater prevalence in glaucoma observed in individuals of AD. The purpose of the present study is to determine if the rate and direction of change in ALCSD due to glaucomatous remodeling differs between patients of AD and ED in the African Descent and Glaucoma Evaluation Study (ADAGES) and the Diagnostic Innovations in Glaucoma Study (DIGS).

## Methods

Participants for this study were recruited from the National Eye Institute–funded University of California at San Diego (UCSD)-based DIGS and the three site multicenter ADAGES (registered at clinicaltrials.gov registered under NCT00221897) that is based at UCSD, Columbia University, and the University of Alabama at Birmingham (UAB). DIGS is conducted only at UCSD and is the longest running structural imaging and functional testing cohort of glaucoma subjects. The current study examined data from 177 eyes from 122 subjects with glaucoma (53 ED and 69 AD) that met all longitudinal image quality requirements with at least six SDOCT imaging examinations along with meeting all other study eligibility criteria outlined below. Longitudinal data from one patient included in the prior baseline analysis^20^ did not pass the study design quality-control criteria and this subject was removed from the current analysis. All participants gave written informed consent. The institutional review boards at all three sites approved the study methods. All methods adhere to the tenets of the Declaration of Helsinki and to the Health Insurance Portability and Accountability Act.

Details of the ADAGES and DIGS studies are described previously.^21^ In brief, ADAGES and DIGS follow identical protocols in which patients with open angle glaucoma are followed every 6 months with a comprehensive eye examination, imaging, and visual field (24-2 Swedish Interactive Thresholding Strategy testing every 6 months). The current study utilizes Spectralis (Heidelberg Engineering, Heidelberg, Germany) SD-OCT imaging data for baseline and follow-up visits since 2010. Details of the inclusion and exclusion criteria are described in detail in the ADAGES baseline study.^21^ Glaucoma was confirmed at baseline with two reliable visual fields at baseline. The UCSD Visual Field Assessment Center reviewed all visual fields for quality throughout the study.^21^

### Image Acquisition and Processing

Imaging was performed approximately every 6 months throughout the study. A Spectralis SD-OCT scan (software version 5.6.4.0 or higher) of the ONH was performed using 48 radial averaged B-scans (15 scans/section) using enhanced depth imaging to assess ALCSD and a high-density retinal nerve fiber layer circle scan was used to assess choroidal thickness.^20^ The San Diego Automated Layer Segmentation Algorithm (SALSA) was used to automatically identify^22^^–^^24^ Bruch's membrane opening (BMO) reference plane, anterior laminar surface depth, and choroidal thickness in each b-scan. SALSA uses a shape-constrained surface evolution method where the surface is refined iteratively using a nonlocal Markov random field-based segmentation. Specifically, the method utilizes both 2D and 3D information of the lamina to overcome the problem of low signal in some areas. The accuracy of the BMO and ALCSD segmentation was reviewed qualitatively. To compute ALCSD, the delineated surfaces were projected onto Cartesian coordinates based on the internal scaling factors provided by the optical coherence tomography (OCT) control software, which utilizes an internal calibration model accounting for imaging distance and focus. ALCSD measurements for the analysis were obtained by averaging ALCS points from all 48 B-scans within a 3-pixel radius (approximately 18 microns) of the BMO centroid.

**Figure 1.**
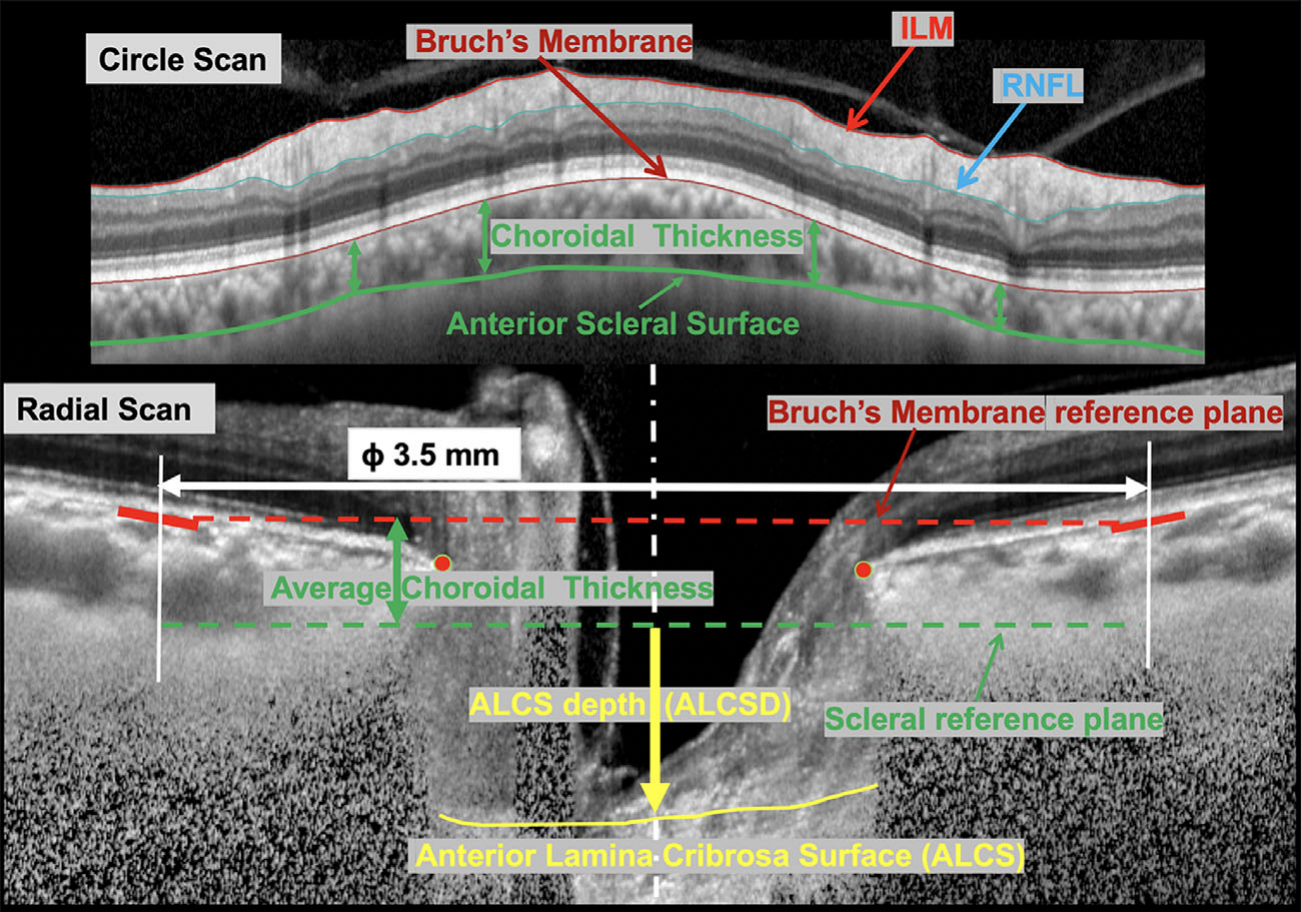
Illustration of the anterior laminar cribrosa surface depth (ALCSD) computation approach. From the circle scan of each eye (upper B-scan image) the internal limiting membrane (ILM), retinal nerve fiber layer (RNFL), Bruch's membrane (BM), and anterior scleral surface are computed by SALSA autosegmentation. Choroidal thickness was computed from the circle scan as the average distance between the BM and the anterior scleral surface. The sclera reference was computed as a plane parallel to the BM reference plane and distant in magnitude equal to the average choroidal thickness (computed from the circle scan). ALCSD was then defined as the average vertical distance between the sclera reference plane and points all the lamina falling within a 3 pixel-radius from the BMO centroid projection on the anterior lamina surface.

SALSA was also used to calculate choroidal thickness from high density (100 ART) retinal nerve fiber layer circle scans (3.5 mm diameter).^24^ Because BMO position may shift with age-related thinning of the choroid and also differs across AD and ED groups,^25^ measurements of ALCSD presented in this study were computed based on a scleral reference plane, as previously described^6^ and illustrated in Figure 1. Briefly, from the circle scan of each eye, choroidal thickness is computed as the average distance between the Bruch's membrane (BM) and the choroid-sclera interface as autosegmented by SALSA. A cylinder, 3.5 mm in diameter oriented along a vertical axis passing for the BM's opening points’ (BMO) centroid, intersects the BM surface in each B-scan of the radial scan. The BM reference plane is computed as the best fitting planed passing through the BM intersection points. The sclera reference plane is then computed as the vertical shift of the BM reference place of the average choroidal thickness computed from the circle scan. Ultimately, in a given B-scan, laminar was defined as the average vertical distance between the sclera reference plane and points all the lamina falling within a 3 pixel-radius from the BMO centroid projection on the anterior lamina surface. The mean ALCSD measurement from all 48 B-scans was used in the analysis. Last, we also performed a secondary analysis using a BMO-based reference plane, as this is a commonly used approach, and provided as an appendix.

### Statistical Analysis

Demographic and baseline ocular characteristics across racial groups were compared by two-sample *t*-tests and Fisher's exact tests for continuous and categorical variables respectively and at patient level, and a linear mixed effects (LME) model with a random patient intercept for eye-level characteristics. For each model, a patient-level random intercept was included to account for correlations within patients across eyes, and within each eye, and an additional random intercept and slope was included to account for temporal correlation within eyes. Additionally, we refit all our mixed models using only one randomly selected eye per patient to ensure these effects were addressed. Associations between baseline ALCSD and ocular and demographic characteristics were determined using a univariable and multivariable LME. Independent variables in the model defining ALCSD included age, race, IOP, central corneal thickness (CCT), BMO, axial length, and visual field mean deviation (VF MD). Similarly, longitudinal changes in ALCSD were assessed with LME. These models included baseline age, follow-up, the independent variables outlined in the baseline analysis, and their interaction with follow-up as covariates, with random intercepts and slopes within eye, and additional patient-level random intercept. All statistical analysis was performed in R Statistics (Foundation for Statistical Computing, Vienna, Austria) and a *P* value of < 0.05 was considered statistically significant.

## Results

**Figure 2.**
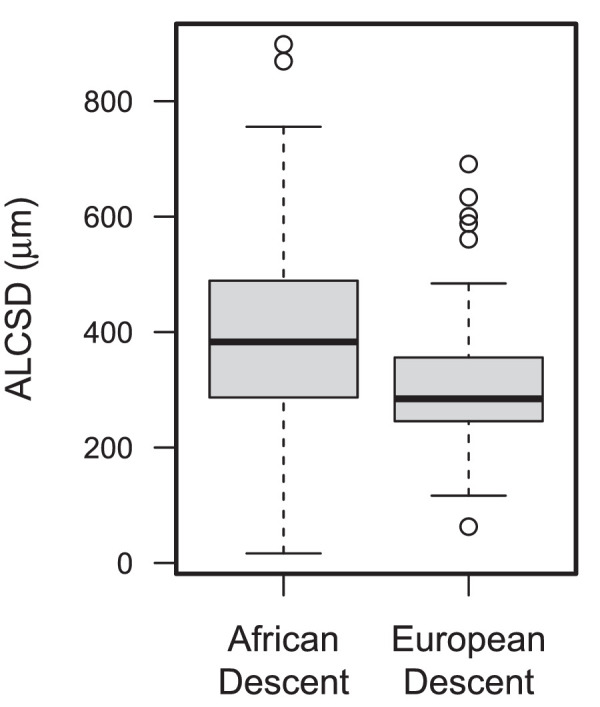
Box and whisker plot of anterior laminar cribrosa surface depth measurements (ALCSD) in patients with glaucoma of African and European descent.

The *t*-test and Fisher's test for ocular, demographic, and study follow-up data are provided in Table 1. Compared to the ED group, the AD group had a lower mean age (4.9 years, *P* = 0.014) and 79.6 µm deeper mean ALCSD (*P* = 0.003; see Fig. 2). All other demographic and ocular parameters were similar across racial groups (*P* > 0.05). The mean follow-up was slightly longer in the AD group (3.2 vs. 2.9 years, *P* = 0.042; see Table 1) and the IOP slightly higher than the ED group (14.9 vs. 13.8 mm Hg, *P* = 0.092; see Table 1). An average of 7.2 images per eye (range = 3 to 11) over a mean follow-up of 3.1 years (range = 1.0 – 4.1 years) were recorded for a total of 1122 scans.

**Table 1.** Patient and Ocular Characteristics by Race

	African Descent	European Descent	
	*n* = 69 (97 Eyes)	*n* = 53 (80 Eyes)	*P* Value
Age	68.3 (65.3 to 71.3)	73.2 (70.6 to 75.8)	**0.014**
Sex			
F	41 (59.4%)	28 (52.8%)	0.587
M	28 (40.6%)	25 (47.2%)	
ALCSD, µm	388.3 (354.7 to 421.9)	308.7 (270.7 to 346.8)	**0.003**
VF MD, dB	−7.48 (−9.06 to −5.91)	−7.06 (−8.83 to −5.30)	0.729
SE, D	−0.8 (−1.2 to −0.4)	−0.3 (−0.8 to 0.1)	0.118
AL, mm	24.0 (23.7 to 24.3)	23.9 (23.6 to 24.2)	0.764
Disc area, mm^2^	2.13 (2.01 to 2.25)	2.09 (1.96 to 2.23)	0.666
IOP, mm Hg	14.9 (14.0 to 15.7)	13.8 (12.8 to 14.7)	0.092
Maximum IOP, mm Hg	17.5 (16.5 to 18.4)	16.0 (14.9 to 17.0)	**0.041**
CCT, µm	530.3 (520.9 to 539.6)	538.7 (528.0 to 549.3)	0.248
Choroid thickness, µm	141.8 (127.5 to 156.0)	129.8 (113.7 to 145.8)	0.276
No. of visits	7.2 (6.7 to 7.7)	7.1 (6.6 to 7.7)	0.811
Follow-up, y	3.2 (3.0 to 3.4)	2.9 (2.7 to 3.1)	**0.042**

Results are presented as mean (95% confidence interval) or count (percentage). Sex was compared using Fisher's exact test. Continuous variables were compared using two-sample *t*-tests (for age) or linear mixed models (for eye level data).

ALCSD, anterior lamina cribrosa surface depth VF MD, visual field mean deviation; SE, spherical equivalent; AL, axial length; IOP, intraocular pressure; CCT, central corneal thickness.

Bold *P* values are significant.

The univariable associations of demographic and ocular characteristics with baseline ALCSD are shown in Table 2. At baseline, shallower ALCSD was associated with age (−2.8 µm/year, *P* = 0.016). ALCSD was shallower in the ED group (−83.74 µm, *P* = 0.003), and was 70.8 µm deeper in men compared to women (*P* = 0.008). Deeper ALCSD was associated with worsening VF at baseline (VFbase; −3.0 µm/1 dB, *P* = 0.027). Maximum historical IOP was significantly associated with deeper ALCSD (5.37 µm/mm Hg, *P* = 0.034), whereas a similar trend for baseline ALCSD and baseline IOP (IOPbase) (magnitude of 5.49 µm/mm Hg) did not reach statistical significance (*P* = 0.07).

**Table 2.** Univariable Models Associating Anterior Lamina Cribrosa Surface Depth at Baseline With Each Variable Of Interest

	Baseline ALCSD, Univariable Models	
	Estimate (95% CI)	*P* Value
Age	−2.80 (−5.06 to −0.55)	**0.016**
Gender: M	70.81 (19.42 to 122.20)	**0.008**
European descent	−79.56 (−130.32 to −28.81)	**0.003**
VFbase	−3.00 (−5.63 to −0.36)	**0.027**
SE	−3.43 (−15.46 to 8.60)	0.577
AL	18.47 (−5.38 to 42.31)	0.131
Disc area	54.84 (8.50 to 101.19)	**0.022**
IOPbase	5.49 (−0.42 to 11.40)	0.070
Maximum IOP	5.37 (0.46 to 10.29)	**0.034**
CCT	−0.01 (−0.65 to 0.63)	0.979

VFbase, visual field mean deviation at baseline; SE, spherical equivalent; AL, axial length; IOP, intraocular pressure; IOPbase, intraocular pressure at baseline; CCT, central corneal thickness.

Bold *P* values are significant; bold italic indicate approaching significance.

**Figure 3.**
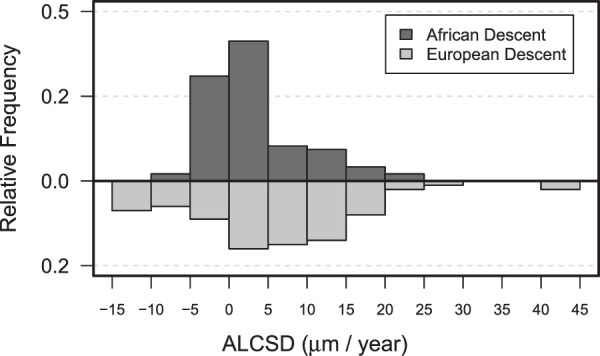
Frequency histogram of the rates of progressive change in anterior laminar cribrosa surface depth measurements (ALCSD) in patients with glaucoma of African and European descent.

The univariable associations of demographic and ocular characteristics with longitudinal measures of ALCSD are shown in Table 3. The estimates for the univariable models in Table 3 are drawn from LME models reporting the variable of interest as main effect (fixed) plus their interaction term with time. The main effects for age (ALCSD = 2.76 µm shallower per year, *P* = 0.007), gender (ALCSD = 59.92 µm deeper in men, *P* = 0.009), and race (ALCSD = 83.7 µm shallower in the ED group, *P* < 0.001) were significant (see Table 3: Main Effects). However, only the time interaction term with race was significant, with the ED group showing significantly greater deepening of ALCSD over time (2.55 µm/year, *P* = 0.037; see Table 3: Time Interaction). The mean of change in ALCSD was 3.01 (95% confidence interval [CI] = 1.45–4.57) in the AD group and 5.66 (95% CI = 3.77–7.35). The distribution of the ALCSD rate of change over time (slope) appeared to be wider in the ED subjects compared to AD subjects (Fig. 3). Although there was overall a greater posterior shift in the position of the lamina cribrosa among ED subjects over time, there were also more ED patients that experienced both deepening and shallowing in ALCSD than in the AD group, as qualitatively shown in the frequncy plot in Figure 3.

**Table 3.** Main Effect and Interaction Estimates From Longitudinal Linear Mixed Models Associating Anterior Lamina Cribrosa Surface Depth With (A) Each Variable of Interest and Time Included as Independent Variables and (B) Each Variable of Interest, Time, and Their Interaction Included as Independent Variables

	Longitudinal ALCSD, Univariable Models			
	(A) Main Effect	*P* Value	(B) Time Interaction	*P* Value
Age	−2.76 (−4.73 to −0.78)	**0.007**	−0.01 (−0.12 to 0.10)	0.842
Gender: M	59.92 (15.45 to 104.40)	**0.009**	−0.13 (−2.53 to 2.26)	0.913
Race: European descent	−83.74 (−127.37 to −40.11)	**<0.001**	2.55 (0.18 to 4.93)	**0.037**
VFbase	0.12 (−0.23 to 0.48)	0.503	−0.04 (−0.18 to 0.11)	0.632
SE	−0.37 (−12.91 to 12.17)	0.954	0.37 (−0.28 to 1.03)	0.264
AL	12.72 (−8.82 to 34.26)	0.249	−0.30 (−1.45 to 0.86)	0.615
Disc area	63.96 (18.72 to 109.20)	**0.006**	−0.83 (−3.37 to 1.71)	0.523
IOP	0.01 (−0.28 to 0.30)	0.955	0.06 (−0.16 to 0.29)	0.579
Maximum IOP	7.00 (1.78 to 12.23)	**0.009**	−0.05 (−0.33 to 0.23)	0.715
CCT	0.12 (−0.44 to 0.67)	0.685	0.01 (−0.02 to 0.04)	0.538

VFbase, visual field mean deviation at baseline; SE, spherical equivalent; AL, axial length; IOP, intraocular pressure; IOPbase, intraocular pressure at baseline; CCT, central corneal thickness.

Bold *P* values are significant.

The multivariable associations of demographic and ocular characteristics with longitudinal measures of ALCSD are shown in Table 4. Only the variables that showed statistically significant association in the univariable models were added to the multivariate model. Adjustment for VF and CCT was kept despite their lack of significant association as univariate to reduce unaccounted mediation effects.

**Table 4.** Longitudinal Multivariate Model Associating Anterior Lamina Cribrosa Surface Depth With All the Variables of Interest With Significant Association as Univariables (See Table 3)

	Estimate	Std. Error	95% CI	*P* Value
(Intercept)	453.84	185.41	(90.45 to 817.23)	**0.016**
Age	−2.14	1.10	(−4.30 to 0.02)	0.054
Gender: M	70.48	25.17	(21.15 to 119.82)	**0.006**
Race: European descent	−61.41	25.68	(−111.74 to −11.08)	**0.018**
VF	0.12	0.18	(−0.23 to 0.47)	0.502
Disc area	49.71	22.28	(6.05 to 93.38)	**0.027**
Maximum IOP	4.34	2.46	(−0.47 to 9.16)	0.079
CCT	−0.24	0.31	(−0.85 to 0.37)	0.441
Time, y	3.01	0.80	(1.45 to 4.57)	**<0.001**
Time, y, X Race: European descent	2.57	1.21	(0.20 to 4.94)	**0.035**

VF, visual field mean deviation; SE, spherical equivalent; AL, axial length; IOP, intraocular pressure; CCT, central corneal thickness.

Bold *P* values are significant; bold italics indicate approaching significance.

In the multivariate model, all the variables maintained the same associative trend shown in their univariate models in Table 3. Of interest, the interaction between race and time was similar to the univariate model with 2.57 µm/year difference between the ED and AD groups (*P* = 0.035), with the ED group showing the faster rate of ALCSD deepening over time.

Performing multivariable analyses using ALCSD based on a BMO reference plane provided similar associations with race when choroidal thickness was adjusted for in the analysis (2.59 microns/year deeper in the ED group, *P* = 0.034). When ALCSD was computed using a BMO-based reference plane,^6^ all associations were similar when choroidal thickness is included in the model (Supplementary Table S5). Last, the models refit using only one randomly selected eye per patient provided similar results as our full models (Supplementary Tables S6, S7).

## Discussion

The current study performed in the ADAGES and DIGS cohorts demonstrates racial differences in the change over time in the depth of the lamina cribrosa between AD and ED patients with glaucoma. There were larger magnitudes of migration of the lamina cribrosa in ED patients over time. On average, during the 3 years of follow-up, there was a greater posterior shift of the ALCSD in ED patients compared with AD patients, despite a shallower average ALCSD in the ED group. These differences were independent of known racial differences in optic disc size measured as BMO area. Although prior longitudinal studies have examined the effect of glaucomatous remodeling on ALCSD in Asian^3^^,^^4^ and European-derived^5^ populations, no prior study has compared changes in ALCSD between AD and ED patients with glaucoma longitudinally. These findings imply that there are racial differences in the remodeling response of the load bearing connective tissues of the optic nerve. In part, this may be related to the differential susceptibility to glaucomatous injury and progression between these two racial groups.

Remodeling of the lamina cribrosa is a well-described component of the glaucomatous process and has been studied in primate models,^26^^–^^33^ human donor tissues,^34^^–^^37^ and more recently in vivo imaging studies.^3^^,^^38^^,^^39^ A shift in the position of the lamina has been demonstrated preceding retinal nerve fiber layer (RNFL) loss in the chronically elevated IOP nonhuman primate model.^29^^,^^40^ In vivo imaging studies in humans have shown that the ALCSD can be affected by the stage of the disease and may potentially remodel to migrate toward a more anterior or posterior position within the scleral canal.^3^

Computational studies suggest that IOP-induced deformation of the ONH can result in posterior displacement by the direct effect of IOP or anterior displacement due to IOP-induced scleral canal expansion, which pulls the lamina cribrosa taut within the canal.^41^^,^^42^ Moreover, the strain experienced within the lamina cribrosa in response to changes in IOP is modulated by the rigidity of the sclera.^41^^,^^42^ For example, there is less canal expansion and more posterior deformation with a stiffer sclera. These observations have been supported in animal models.^43^^–^^45^ Interestingly, we have previously demonstrated that scleral structural stiffness differs between AD and ED racial groups and that age-related sclera stiffening was more pronounced in eyes from AD organ donors.^46^^,^^47^ These differences are likely related to the observed racial differences in the mechanical behavior of the lamina cribrosa acutely^6^ that drives long-term connective tissue remodeling.

Several cross-sectional studies have demonstrated associations between ALCSD remodeling and both glaucoma severity and aging.^3^^,^^20^^,^^38^^,^^39^^,^^48^ In subjects without eye disease, we have previously demonstrated racial differences in the association between ALCSD and normal aging, with a deeper ALCSD associated with increasing age in the AD group compared to the ED group.^19^ These differences in remodeling due to normal aging across these racial groups parallel the associations we have observed in quantifications of three-dimensional episcopic reconstructions of the human ONH from eye donors.^49^ Specifically, we found an increasing depth of the lamina cribrosa surface and thickening of the sclera was associated with increasing age in eyes from AD donors.^39^

In patients with glaucoma, Lee et al. and Park et al. demonstrated that ALCSD migrated posteriorly (deepened) with increasing disease severity.^3^^,^^38^^,^^39^ In a cross-sectional study of an ED population of patients with high-risk ocular hypertensive and glaucoma, Ren and colleagues demonstrated an age dependent effect on remodeling of the lamina cribrosa with deeper ALCSD in younger eyes and shallower ALCSD in older eyes in models adjusted for disease severity; specifically ALCSD deepened in eyes with increasing glaucoma severity in younger eyes but not in older eyes.^48^ Last, in a prior ADAGES/DIGS cross-sectional study of baseline SD-OCT data, we also found that a deeper ALCSD was associated with increasing glaucoma severity, increasing IOP, male gender, and African descent.^20^ In this group of patients with glaucoma, we found an interaction with aging and race such that the AD group exhibited a more shallow ALCSD with older age after adjusting for disease severity compared to the ED group. These studies suggest a complex relationship between lamina displacement due to normal aging and due to glaucoma that also varies across these racial groups with differential susceptibility to glaucomatous injury.

There have been a limited number of longitudinal studies examining ALCSD in glaucoma^3^^–^^5^ and none to our knowledge that have included both individuals of African and European Descent, groups with known differences in optic nerve biomechanics.^6^ Although prior cross-sectional studies showed that increasing glaucoma severity was associated with a deeper lamina cribrosa, three recent longitudinal studies of laminar depth showed both anterior or posterior displacement of ALCSD over time in Asian and European-descent patients.^3^^–^^5^ Each of these studies demonstrated a broad distribution of anterior and posterior shifts in ALCSD. (Wu et al.^3^ 12% anterior vs. 12% posterior, Wong et al. 15% anterior vs. 11.5% posterior, and Kim et al. 37% anterior vs. 35% posterior). Whereas we have reported ALCSD change as a continuous slope, our findings confirm these prior studies with ALCSD showing both anterior and posterior migration (see Fig. 2). Moreover, this is the first study to demonstrate the independent effect of race on longitudinal change in ALCSD.

The results of our current study provide longitudinal confirmation of our previously reported cross-sectional associations between disease severity and ALCSD in the ADAGES cohort.^20^ These findings are in contrast to prior cross-sectional studies of the effects of age-related changes in ALCSD,^19^ which demonstrated a deeper ALCSD associated with advancing age in the AD population. This discrepancy suggests that race may impact age- and glaucoma-related remodeling of the lamina cribrosa differently, with greater age-related posterior migration of the ALCSD in the AD group in contrast to greater posterior glaucomatous remodeling in the ED group. Larger longitudinal studies in healthy subjects in these racial groups are needed to validate these cross-sectional differences.

This prospectively designed multicenter cohort study has several strengths. All imaging was conducted with highly trained technicians and images were quantified using three-dimensional automated segmentations, not single B-scans like some of the prior work. There also are several limitations. There was a difference in age between the racial groups, with an older and broader range of ages in the ED group. Although all the analyses were age-adjusted, it is possible the lack of association in the longitudinal change in ALCSD and age may be due to these differences. However, we have previously demonstrated that the associations of deeper ALCSD with increasing age within the baseline data were all similar using the full dataset or a truncated dataset obtained matching for age.^20^ As discussed in previous ADAGES publications, there are limitations with self-described racial categorization.^21^ However, we have shown that biogeographical ancestry testing correlated strongly with self-described race and that genetic ancestral testing added no additional information regarding known racial differences in ocular structures (corneal thickness and optic BMO area) beyond self-described race alone.^50^

In summary, the current study demonstrated that glaucomatous remodeling changes in the position of the lamina cribrosa may differ between ED and AD racial groups. The greater posterior shift in ALCSD seen in the ED group is in contrast to the changes reported with normal aging, and indicates that there may be a differential effect across these racial groups in remodeling of the optic nerve head. Although these racial differences in remodeling may explain, in part, the differential susceptibility to glaucomatous injury observed in populations of African descent, additional follow-up is needed to define the relationship between changes in the supportive load bearing connective tissues of the ONH and the progressive injury to the overlying neurovascular tissues they support.

## Supplementary Material

Supplement 1
