# Supplementary material for: Racial Differences in the Rate of Change in Anterior Lamina Cribrosa Surface Depth in the African Descent and Glaucoma Evaluation Study
Source: Invest Ophthalmol Vis Sci. 2021 Apr 12;62(4):12. doi: 10.1167/iovs.62.4.12 (PMC8039570; doi:10.1167/iovs.62.4.12)
Supplement: Supplement 1 [file iovs-62-4-12_s001.pdf]

Table 5 (appendix): Longitudinal multivariable linear mixed model for Anterior Cribrosa Surface Depth defined by Bruch's Membrane Opening over time adjusting for choroidal thickness

	Estimate	Std. Error	95% CI	p-value
(Intercept)	431.88	161.36	(115.62, 748.14)	**0.008**
Age	-2.75	1.03	(-4.77, -0.72)	**0.009**
Gender: Male	67.87	22.05	(24.67, 111.08)	**0.002**
Race: European Descent	-68.27	22.62	(-112.61, -23.92)	**0.003**
VF	0.12	0.18	(-0.23, 0.47)	0.507
Disc Area	58.18	21.56	(15.91, 100.44)	**0.008**
Maximum IOP	6.09	2.66	(0.88, 11.30)	**0.023**
CCT	-0.12	0.28	(-0.67, 0.43)	0.672
Time (Years)	0.70	0.18	(0.35, 1.06)	**<0.001**
Time (Years) X Race: European Descent	3.00	0.80	(1.44, 4.57)	**<0.001**

Abbreviations: ALCSD, anterior lamina cribrosa surface depth VF MD, visual field mean deviation; SE, spherical equivalent; AL, axial length; IOP, intraocular pressure; CCT, central corneal thickness

Table 6 (appendix): Main effect and interaction estimates from longitudinal linear mixed models associating anterior lamina cribrosa surface depth (ALCSD) using only one randomly selected eye per patient with a) each variable of interest and time included as independent variables and b) each variable of interest, time, and their interaction included as independent variables.

**Longitudinal ALCSD, univariable models**				
	**A) Main Effect**	**p-value**	**B) Time Interaction**	**p-value**
Age	-2.91 (-5.12, -0.69)	**0.011**	0.00 (-0.13, 0.14)	0.951
Gender: Male	73.77 (23.16, 124.39)	**0.005**	-0.15 (-3.15, 2.85)	0.922
Race: European Descent	-62.96 (-114.08, -11.83)	**0.017**	3.08 (0.12, 6.04)	**0.044**
VFbase	0.32 (-0.17, 0.80)	0.204	-0.02 (-0.21, 0.17)	0.839
SE	-4.29 (-18.79, 10.22)	0.564	0.25 (-0.58, 1.08)	0.557
AL	24.40 (0.21, 48.60)	0.050	0.23 (-1.19, 1.65)	0.751
Disc Area	64.63 (15.43, 113.84)	**0.011**	-0.02 (-3.08, 3.03)	0.987
IOP	-0.08 (-0.43, 0.26)	0.637	0.08 (-0.20, 0.35)	0.571
Maximum IOP	6.66 (0.73, 12.58)	**0.030**	-0.03 (-0.38, 0.31)	0.848
CCT	-0.04 (-0.70, 0.61)	0.899	0.01 (-0.02, 0.05)	0.516

Abbreviations: VFbase, visual field mean deviation at baseline; SE, spherical equivalent; AL, axial length; IOP, intraocular pressure; IOPbase, intraocular pressure at baseline; CCT, central corneal thickness. Bold p-values are significant

Table 7 (appendix): Longitudinal multivariate model associating anterior lamina cribrosa surface depth (ALCSD) using only one randomly selected eye per patient with all the variables of interest with significant association as univariables (see Table 3).

	**Estimate**	**Std. Error**	**95% CI**	**p-value**
(Intercept)	502.43	191.93	(126.26, 878.60)	**0.010**
Age	-2.48	1.09	(-4.62, -0.33)	**0.025**
Gender: Male	69.85	25.03	(20.80, 118.90)	**0.006**
Race: European Descent	-41.14	25.68	(-91.46, 9.19)	0.112
VF	0.32	0.25	(-0.17, 0.81)	0.196
Disc Area	59.2	23.66	(12.83, 105.58)	**0.014**
Maximum IOP	5.78	2.94	(0.03, 11.53)	0.051
CCT	-0.38	0.32	(-1.01, 0.25)	0.238
Time (Years)	2.75	0.98	(0.84, 4.67)	**0.006**
Time (Years) X Race: European Descent	3.14	1.5	(0.19, 6.09)	**0.039**

Abbreviations: VF, visual field mean deviation; SE, spherical equivalent; AL, axial length; IOP, intraocular pressure; CCT, central corneal thickness. Bold p-values are significant; bold talic approaching significance.
